# Ranaviruses Bind Cells from Different Species through Interaction with Heparan Sulfate

**DOI:** 10.3390/v11070593

**Published:** 2019-06-29

**Authors:** Fei Ke, Zi-Hao Wang, Cheng-Yue Ming, Qi-Ya Zhang

**Affiliations:** 1 State Key Laboratory of Freshwater Ecology and Biotechnology, The Innovation Academy of Seed Design, Institute of Hydrobiology, Chinese Academy of Sciences, Wuhan 430072, China; 2 College of Modern Agriculture Sciences, University of Chinese Academy of Sciences, Beijing 100049, China

**Keywords:** ranavirus, virus binding, heparan sulfate, *Andrias davidianus* ranavirus, *Rana grylio* virus, envelope protein

## Abstract

Ranavirus cross-species infections have been documented, but the viral proteins involved in the interaction with cell receptors have not yet been identified. Here, viral cell-binding proteins and their cognate cellular receptors were investigated using two ranaviruses, *Andrias davidianus* ranavirus (ADRV) and *Rana grylio* virus (RGV), and two different cell lines, Chinese giant salamander thymus cells (GSTC) and *Epithelioma papulosum* cyprinid (EPC) cells. The heparan sulfate (HS) analog heparin inhibited plaque formation of ADRV and RGV in the two cell lines by more than 80% at a concentration of 5 μg/mL. In addition, enzymatic removal of cell surface HS by heparinase I markedly reduced plaque formation by both viruses and competition with heparin reduced virus-cell binding. These results indicate that cell surface HS is involved in ADRV and RGV cell binding and infection. Furthermore, recombinant viral envelope proteins ADRV-58L and RGV-53R bound heparin-Sepharose beads implying the potential that cell surface HS is involved in the initial interaction between ranaviruses and susceptible host cells. To our knowledge, this is the first report identifying cell surface HS as ranavirus binding factor and furthers understanding of interactions between ranaviruses and host cells.

## 1. Introduction

Interspecies transmission and infection have been reported in several viruses that infect humans [1,2]. Likewise, aquatic animal viruses can also infect and cause disease in a wide range of aquatic animals [3,4]. Specifically, the interspecies infection was reported following ranavirus infection [5,6]. However, the basis for the broad ranavirus host range is not clear [7,8]. Ranaviruses are large double-stranded DNA viruses within the family *Iridoviridae* [9]. Ranaviruses target aquatic animals globally and have been isolated from reptiles [10,11], amphibians [12,13,14,15], and bony fish [16,17]. Among them, several isolates represent great threats to the development of the aquaculture industry and wild animal populations [18]. Aside from infecting several species within a given taxonomic class (e.g., *frog virus 3* infects diverse amphibian species), ranaviruses may also infect members of different classes (e.g., *frog virus 3*-like agents have been isolated from both amphibians and fish) [19].

Binding susceptible host cells is the first step in viral infection and is one of the key factors responsible for determining host range [20]. Diverse cellular receptors and viral envelope and capsid proteins are involved in the process. For example, vaccinia virus utilizes four viral proteins that bind to glycosaminoglycans (GAGs) or laminin on the cell surface [21,22,23,24,25].

Cell surface GAGs consist of complex linear polysaccharides, which are ubiquitously expressed in most cell types [26]. Heparan sulfate (HS), chondroitin sulfate (CS), and dermatan sulfate comprise the main types of GAGs on the cell surface. Besides vaccinia virus, GAGs are involved in the binding of adenovirus [27], various alphaviruses [28,29], bunyaviruses [30,31], filoviruses [32], flavivirus [33], hepacivirus [34], herpesvirus [35], papillomaviruses [36], and rhabdoviruses [37]. It has been shown that cell surface GAGs, especially HS, serve as initial receptors in infections with these viruses. However, there is little information on the role of HS in ranavirus binding.

*Rana grylio* virus (RGV) and *Andrias davidianus* ranavirus (ADRV) are ranaviruses isolated from diseased pig frogs *R. grylio* (anura amphibian) and Chinese giant salamanders (CGS) *A. davidianus* (urodele amphibian), respectively [12,13]. The complete genomes of the two viruses have been sequenced, and several functional proteins have been characterized [38,39,40,41,42,43,44,45]. For example, RGV-53R, a homolog of ADRV-58L, was identified as an envelope protein [38]. Decreased expression of RGV-53R, or its *frog virus 3* (FV3) homolog FV3-53R impaired virus replication in cultured cells [45,46]. In this report, we examine the role of HS in ranavirus entry. Furthermore, since ranavirus viral envelope proteins [38,39,40,45,46,47,48] are likely involved in the initial interaction between virus and host, we examined the ability of RGV-53R and its ADRV homolog, ADVR-58L, to bind heparin-Sepharose beads.

## 2. Materials and Methods

### 2.1. Viruses and Cells

ADRV isolated from diseased Chinese giant salamanders (CGS) [13] and RGV isolated from the diseased pig frog *R. grylio* [12] were maintained in our laboratory and used in the present study. CGS thymus cells (GSTC) [49] and *Epithelioma papulosum* cyprinid (EPC) cells [39] were cultured in M199 medium supplemented with 10% bovine calf serum.

### 2.2. Virus Purification

ADRV and RGV were purified, as described previously [50]. Briefly, GSTC cells were infected with ADRV and RGV at a multiplicity of infection (MOI) of 0.1 PFU/cell, respectively, and incubated at 25 °C. The cell cultures were harvested when cytopathic effects (CPE) reached approximately 90%. The viral suspensions were frozen at −20 °C, thawed three times, and then centrifuged at 5000× *g* for 20 min. The resulting supernatants were ultracentrifuged at 110,000× *g* (Beckman, SW41, Brea, CA, USA) for 90 min. The pellets were resuspended in TE buffer (10 mM of Tris-HCl, 1 mM of EDTA, pH 7.4) and further purified in a discontinuous sucrose gradient (30%, 40%, 50%, and 60%) at 110,000× *g* for 60 min. The viral bands were collected, centrifuged to remove residual sucrose, and the resulting viral pellets were resuspended in TE buffer and stored at −80 °C.

### 2.3. Plaque Reduction Assay

Heparin is a structural homolog of highly sulfated HS and has been used as a surrogate for cell surface HS in research studies examining binding [51]. The effect of heparin on viral plaque formation was tested in assays using GSTC and EPC cells for the purpose of analyzing the effect of soluble glycosaminoglycans on viral infection. The indicated cells were seeded in 24-well plates 24 h prior to infection. Heparin (Sangon Biotech, Shanghai, China, from porcine intestinal mucosa, molecular weight range of 6–20 kDa) was diluted in cell culture medium and incubated with ADRV or RGV for 1 h at 4 °C. At a concentration of 10 μg/mL, the color of the growth medium (using phenol red as an indicator) did not change, indicating a stable pH. Cell culture media was removed and 100 μL of the virus-heparin solution, containing approximately 50 PFU of the indicated virus, was added to each well for 1 h at 25 °C. Three replicate wells were used in each treatment. After 1 h, the inoculum was removed, the cells were washed twice with fresh medium, and overlaid with culture media containing 0.7% agarose. Fresh medium was added to the well after agarose solidification. Plaques were counted after three days of incubation at 25 °C. Based on the results obtained from the heparin treatment, two other glycosaminoglycans, heparan sulfate and chondroitin sulfate (Sigma, St. Louis, MO, USA), were tested by the method described above.

### 2.4. Heparinase Treatment

To further investigate its role in virus binding, target cells were treated with heparinase to remove cell surface heparan sulfate. Heparinase I cleaves the linkages between hexosamines and the O-sulfated iduronic acids of heparin and HS. GSTC cells were seeded in 24-well plates 24 h prior to infection. Medium was removed before the assay, and the cells were incubated with different concentrations of heparinase I from *Flavobacterium heparinum* (Sigma) in 20 mM of Tris-HCl (pH 7.5), 4 mM of CaCl_2_, 50 mM of NaCl, and 0.01% bovine serum albumin (BSA) for 1 h at 15 °C and then washed twice with fresh medium. One hundred microliters of medium containing ADRV or RGV (50 PFU) were added and incubated for another 1 h at 15 °C. The supernatant was removed, the cells washed twice with fresh medium, and overlaid with medium containing 0.7% agarose, as described above. After incubation for three days at 25 °C, the plaques were counted.

### 2.5. Cell Binding Assay

To investigate the effect of heparin on virus–cell binding, quantitative real-time PCR (qPCR) analysis that has been used in the detection of hepatitis C virus (HCV) genomes [34] was used to determine the relative quantity of virus bound to the cell surface. Both purified virions, isolated following separation on a sucrose step gradient, and a crude viral suspension, obtained by lysis of cells infected by the virus, were used in these assays. GSTC cells were seeded in 24-well plates 24 h prior to infection. Heparin was diluted in medium and mixed with the viral suspension or purified virions for 30 min at 4 °C, and then 100 μL of the mixture containing approximately 1000 PFU of the indicated virus was added to cells for 1 h at 4 °C, as described above. The inoculum was removed after incubation. The cells, washed twice with fresh medium, were collected by centrifugation, and DNA was extracted with the TakaRa MiniBEST Universal Genomic DNA Extraction Kit (TakaRa, Tokyo, Japan). Bound viral genomes, based on detection of the relative numbers of major capsid protein gene (MCP), were detected by qPCR, which was conducted using a StepOne Real-Time PCR system (The Applied Biosystems, Foster City, CA, USA). Each qPCR mixture contained 1 μL of DNA, 12.5 μL of SYBR Premix (2×), 0.5 μL of forward and reversed primers (for each primer, 5′-CACCTCCATCCCAGTCAGCA-3′/5′-AATCCCATCGAGCCGTTCA-3′), and 10.5 μL of ultrapure water. The qPCR conditions were as follows: 95 °C for 10 min; 40 cycles of 95 °C for 15 s and 60 °C for 1 min; and a melt curve analysis at 95 °C for 15 s, 60 °C for 1 min, and 95 °C for 15 s. The β-actin gene, used in a previous study [52], was used as a loading control. qPCR efficiency was evaluated with a standard curve, based on serially diluted DNA samples using the β-actin gene primers, which showed that there were no obvious differences on the qPCR efficiencies among samples treated with different concentrations of heparin. For the MCP detection, MCP levels were normalized to β-actin levels in each sample. The level of bound virus (MCP level) in the treated group versus that in the control group (no heparin) was calculated by the 2^−ΔΔ*C*T^ method [53].

### 2.6. Protein Expression and Purification

Considering the pivotal roles that viral envelope proteins play in virus attachment and entry, we determined whether viral envelope proteins interacted with HS by monitored the binding of purified recombinant proteins to heparin-Sepharose beads as described by Chung et al. [22]. Previous studies demonstrated that RGV-53R is 522 amino acid (aa) envelope protein with two predicted transmembrane (TM) domains (aa 193–211 and aa 218–237) [38]. ADRV-58L is the ADRV homolog of RGV-53R. The aa sequence identity between ADRV-58L and RGV-53R is 99.2% [13]. In the present assay, TM helices and topology of the two proteins were predicted with the online tools HMMTOP (http://www.enzim.hu/hmmtop/html/submit.html) and TMHMM (http://www.cbs.dtu.dk/services/TMHMM-2.0/) that presented on ExPASy. The primers 5′-GTAGAATTCATGGGAGCAGCGGAA-3′ and 5′-GATAAGCTTTTATGTGGTGGGGTCCAGGCC-3′ were used for amplifying DNA sequences encoding the N-terminal region (1–192) of 58L and the N-terminal region (1–192) of 53R, respectively. The other pair of primers (5′-GGCGAATTCCCCAGGCCCGTCAAGA-3′/5′-CTATAAGCTTTTAACCCCTGTGGGC-3′) was used for amplifying the DNA sequences for the C-terminal region (238–522) of 58L. Because the amino acid sequence of the C-terminal regions of ADRV-58L and RGV-53R are identical, only the C-terminal region of ADRV-58L was expressed in the present study. The resulting fragments were digested using EcoR I and Hind III, and ligated into pET32a or pET28a vectors that had been digested with the same enzymes. Successful cloning was validated by DNA sequencing.

For protein expression and purification, the plasmids obtained above were used to transform *Escherichia coli* BL21 (DE3). Positive clones were cultured in LB medium and induced with 0.1 mM isopropyl-β-d-thiogalactopyranoside (IPTG) for 4 h at 24 °C. The bacterial pellets were lysed by sonication. The recombinant protein was purified using the HisBind Purification Kit (Novagen, Billerica, MA, USA) according to the manufacturer’s instructions. The purified protein was dialyzed against PBS, the concentration determined using a BCA Protein Assay Kit (Beyotime, Wuhan, China), and stored at −80 °C.

### 2.7. Heparin-Sepharose Binding Assay

Heparin-Sepharose beads 6FF and control Sepharose beads (Purchased from SMART lifesciences, Changzhou, China, particle diameter range of 45–165 μm) were equilibrated with binding buffer (50 mM Tris-HCl, 10 mM sodium citrate, pH 7.4) before use. The purified recombinant protein (5 μg) was mixed with or without heparan sulfate (100 μg/mL) and incubated in binding buffer with 100 μL of beads for 1 h at 4 °C. The supernatant was collected after centrifugation for 1 min at 1800× *g*. The beads were washed with binding buffer (100 μL) five times, and bound protein was eluted with binding buffer containing 2 M NaCl. The samples were analyzed by 12% SDS-PAGE and subsequently transferred to a PVDF membrane (Millipore, Burlington, MA, USA). A monoclonal antibody against the His tag (Santa Cruz, Dallas, Texas, USA) was used as the primary antibody, horseradish peroxidase (HRP)-conjugated goat anti-mouse IgG (H + L) (Merck, Kenilworth, NJ, USA) as the secondary antibody, and antibody binding detected by chemiluminescence (Millipore).

## 3. Results

### 3.1. Heparin and HS Inhibit Infection by ADRV and RGV

**Figure 1:**
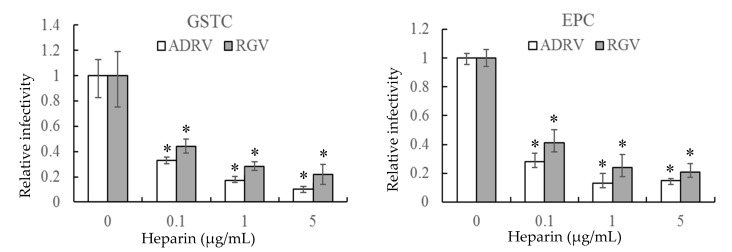
Soluble heparin inhibits *Andrias davidianus* ranavirus (ADRV) and *Rana grylio* virus (RGV) infection of giant salamander thymus cells (GSTC) and *Epithelioma papulosum* cyprinid (EPC) cells. Cells were infected with ADRV or RGV that had been pre-incubated in the presence of different concentrations of heparin. The number of plaques obtained in the absence of heparin was set as 1. The data represent triplicate results and was analyzed with Student’s *t*-test. Significant differences (versus virus without heparin) are marked with * (*p* < 0.05).

The effect of heparin on virus infection was tested by monitoring viral plaque formation following incubation of ADRV and RGV in the presence of increasing concentrations of heparin. As shown in Figure 1, the number of plaques formed by either ADRV or RGV was reduced by heparin in a concentration-dependent manner. For ADRV, infectivity was reduced by approximately 70% by pre-exposure to heparin at 0.1 μg/mL and by more than 80% at 5 μg/mL. A similar phenomenon was observed in RGV-infected cells, which exhibited a 57% inhibition at 0.1 μg/mL and more than 75% at 5 μg/mL. Moreover, inhibition was detected regardless of the cell line used. The results indicate that heparin-like GAGs were involved in ADRV and RGV binding.

**Figure 2:**
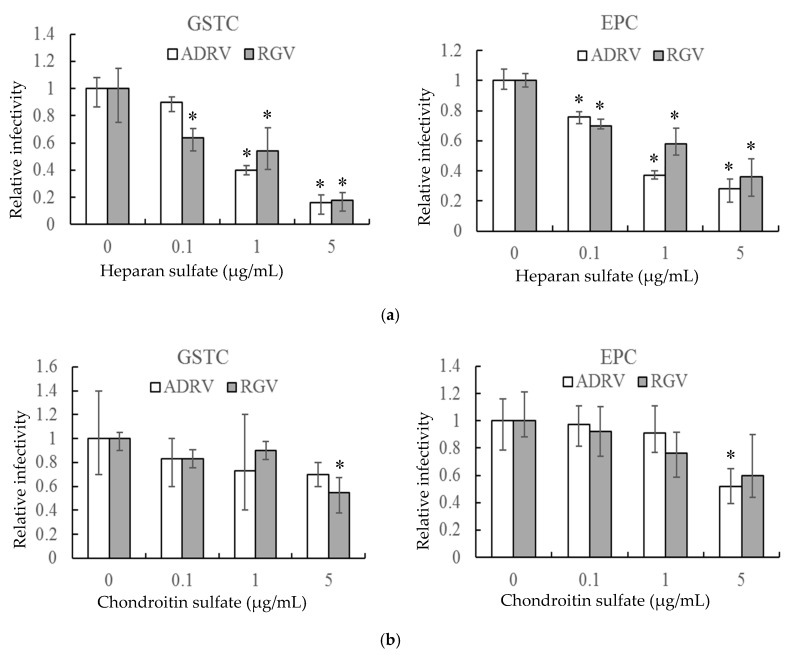
Heparan sulfate (**a**) and chondroitin sulfate (**b**) inhibit ADRV and RGV infection of GSTC and EPC cells. GSTC and EPC cells were infected with ADRV or RGV in the presence of different concentrations of heparan sulfate and chondroitin sulfate. The number of plaques obtained without glycosaminoglycans (GAGs) was set as 1. Experiments were conducted in triplicate and analyzed using Student’s *t*-test. Significant differences (versus virus without exposure to GAGs) are marked with * (*p* < 0.05).

In a second experiment, HS and CS, linear polysaccharides that constitute two major classes of cell surface GAGs, were monitored for their ability to reduce plaque formation. As with heparin, HS reduced the number of plaques formed by the two viruses in GSTC and EPC cells in a concentration-dependent manner (Figure 2a). For both viruses, plaque formation was inhibited by more than 80% in GSTC cells and more than 60% in EPC cells at 5 μg/mL (Figure 2a). In contrast, a significant inhibitory effect was only seen at the highest concentration when CS was substituted for HS (Figure 2b). These results indicate that cell surface GAGs, including HS, likely play important roles in plaque formation by both ADRV and RGV.

### 3.2. Enzymatic Removal of Cell Surface HS Reduced Viral Infection

**Figure 3:**
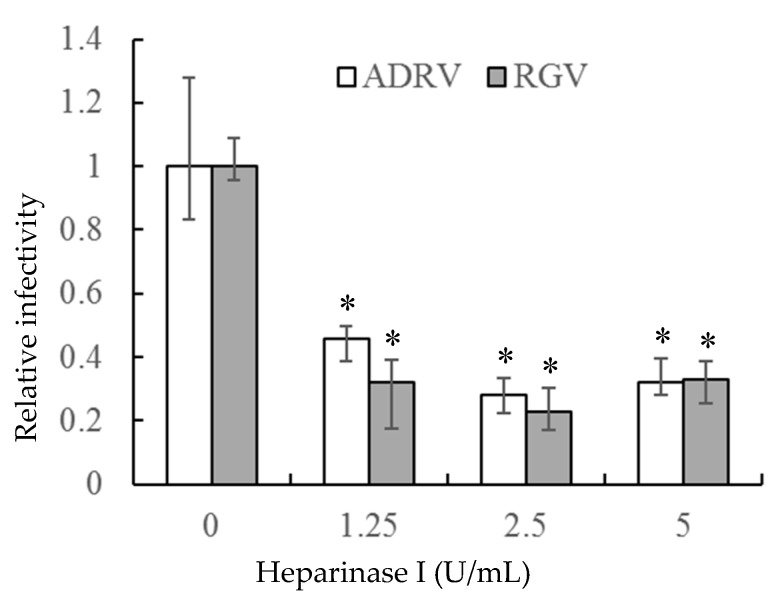
Heparinase I treatment reduced ADRV and RGV plaque formation in GSTC cells. Cells were infected with ADRV or RGV after treatment with different concentrations of heparinase I and plaque formation monitored. Plaque numbers obtained in the absence of heparinase treatment were set as 1. Triplicate results were analyzed by Student’s *t*-test, and significant differences are marked with * (*p* < 0.05).

If the interaction between heparin-like GAGs and viral particles is needed for viral infection, removal of GAGs should inhibit infection. To accomplish that task, heparinase I was used to remove cell surface HS. As shown in Figure 3, the number of plaques formed by the two viruses was markedly reduced in GSTC cells pretreated with heparinase I. The reduction was more than 50% at a heparinase concentration of 1.25 U/mL and slightly more at higher enzyme concentrations. Thus, the ability of heparinase treatment to reduce viral plaque formation supports the view that cell surface HS is a receptor for the two viruses.

### 3.3. Heparin Inhibits Virus–Cell Binding

**Figure 4:**
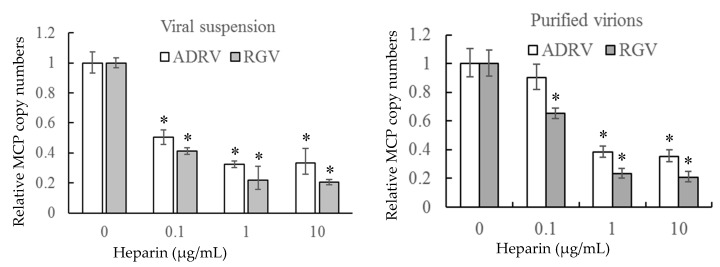
Soluble heparin inhibits virus binding to GSTC cells. Viral suspensions or purified virions were added to GSTC cells in the presence of different concentrations of heparin. After incubation, virion binding was assessed by determining the number of bound viral genomes by qPCR. DNA levels observed in the absence of heparin pre-treatment were set as 1. The data were obtained from three experiments and analyzed with Student’s *t*-test. Significant differences are marked with * (*p* < 0.05).

To further verify the role of cell surface HS on virus–cell binding, heparin was used to inhibit the binding of the two viruses competitively. The number of bound virions was determined by measurement of viral DNA copy number by qPCR to monitor binding. As shown in Figure 4, binding of either crude (viral suspension) or purified virions was inhibited by pre-exposure to heparin. Binding of crude suspensions of either ADRV or RGV were reduced to 20–30% of control levels at a concentration of 10 μg/mL. Similar results were obtained with purified virions. The inhibitory effect of heparin on virus-cell binding supports the finding that cell surface HS is a viral receptor.

### 3.4. Recombinant Envelope Proteins Bind Heparin Beads In Vitro

**Figure 5:**
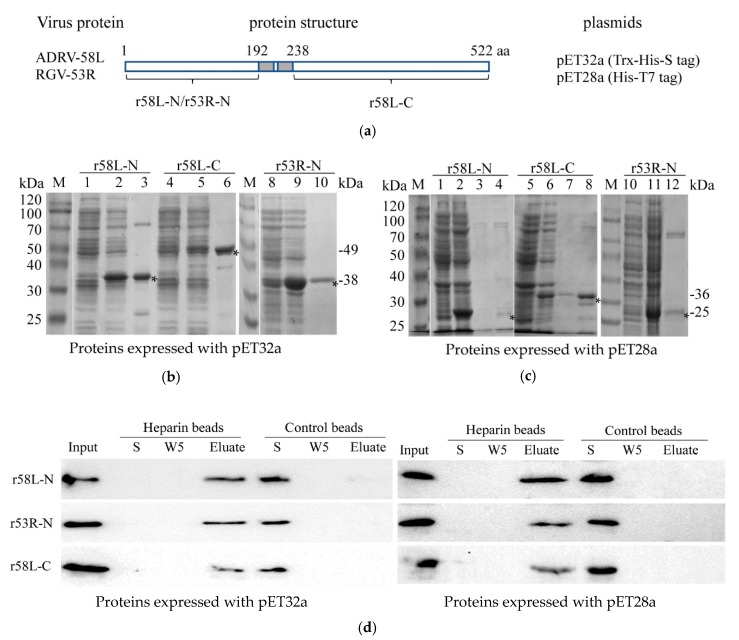
Recombinant proteins bind heparin-Sepharose beads. (**a**) Schematic diagram of the recombinant proteins: The N-terminal domain of ADRV-58L (r58L-N), the N-terminal domain of RGV-53R (r53R-N), and the C-terminal domain of ADRV-58L (r58L-C) were expressed using pET32a or pET28a. The predicted transmembrane region is shown in the grey box. (**b**) Expression and purification of the three proteins (r58L-N, r53R-N, and r58L-C) with pET32a vector. M: protein marker; 1, 4, 8: Bacteria without induction; 2, 5, 9: Bacteria with induction; 3, 6, 10: Purified proteins. The recombinant proteins are indicated with asterisks, and their predicted molecular weights are shown on the right. (**c**) Expression and purification of the three proteins using pET28a. M: Protein marker; 1, 5, 10: Cacteria without induction; 2, 6, 11: Cacteria with induction; 3, 4, 7, 8, 12: Purified proteins. The recombinant proteins are indicated with asterisks, and their predicted molecular weights are shown on the right. (**d**) Binding of recombinant proteins and heparin-Sepharose beads. Recombinant proteins were incubated with heparin-Sepharose or Sepharose beads. The fractions of input (Input), supernatant after incubation (S), the fifth wash solution (W5), and the eluate (Eluate) were detected by Western blot with the anti-His antibody. Recombinant proteins expressed with pET32a or pET28a vectors were used. Recombinant proteins were observed in the Input and Elute fractions from heparin-Sepharose beads and S fraction from control beads.

As shown above, cell surface HS is an important receptor for both ADRV and RGV. Here we examine the role that viral envelope proteins play in this process by monitoring the interaction of purified recombinant viral envelope protein with heparin-Sepharose beads. To accomplish this, the amino terminal 192 amino acids of ADRV-58L and RGV-53R and the C-terminal region of ADRV-58L (amino acids 238–522) were cloned into pET32a and pET28a and expressed in *E. coli* (Figure 5a). Because the amino acid sequences of the C-terminal regions of ADRV-58L and RGV-53R are identical, only the C-terminal region of ADRV-58L was expressed. Note the recombinant proteins were increased in size, due to a 17 kDa Trx-His-S tag in pET32a, and a 4 kDa His-T7 tag in pET28a. As shown in Figure 5b,c, recombinant proteins of the expected sizes were generated using the two expression systems. Subsequently, purified recombinant proteins were isolated and incubated with heparin-Sepharose beads or control beads lacking heparin. The three recombinant proteins (r58L-N, r53R-N, and r58L-C), expressed using either pET32a or pET28a, bound heparin-sepharose beads and were eluted in the presence of a high salt wash. In contrast, all three recombinant proteins failed to bind sepharose beads lacking heparin, and, as a result, were present in the unbound supernatant (S) fraction (Figure 5d). When considered together, these results support the view that HS is a cellular receptor for both ADRV and RGV and the binding fashion may be similar to the interaction between 53R/58L and heparin that occurred in vitro.

## 4. Discussion

In this study, we tested the ability of heparin and two other GAGs (HS and CS) to inhibit plaque formation in fish and amphibian cell lines. These and other results showed that cell surface HS is an important receptor for the binding of ADRV and RGV to target cells. As far as we know, it is the first report describing the role of cell surface HS in iridovirus infection.

Previously we identified RGV-53R as an envelope protein [38]. Here we tested whether RGV-53R, or its ADRV homolog (58L), could bind HS. Our data showed that recombinant 53R and 58L proteins specifically bound heparin-Sepharose beads in vitro and that this binding could be inhibited by the presence of excess HS. The envelope protein 53R and its homologs among other members of the family constitute one of 26 core proteins and likely function in viral entry. Additional studies will be needed to identify the protein domains involved in 53R-heparan sulfate interaction.

Multiple steps are involved in virion entry and initiation of a successful infection. Binding to cell surface GAGs, including HS, has proved to be the initial event in the entry of several mammalian viruses [27,28,29,30,31,32,33,34,35,36,37]. Because GAGs are linked to proteins and usually exist as proteoglycans in vivo [26], viral envelope proteins may bind to cell surface heparan sulfate-linked proteins and facilitate attachment between the virus and potential host cells. Our results suggest that binding of cell surface HS is required for initiation of productive infection by ranaviruses. However, since competition by increasing concentrations of heparin or HS, or treatment with heparinase did not completely inhibit plaque formation, it appears that HS is not the sole cellular receptor for ranaviruses. A similar phenomenon has been observed in the binding of the vaccinia virus, which uses cell surface GAGs and cellular matrix laminin as receptors [22,23,24,25]. A recent study showed that class A scavenger receptors are utilized by *frog virus 3*, the type species of the genus *Ranavirus* [54]. Additional ranavirus binding factors and specific cellular protein receptors may be involved in viral attachment and entry. In addition, although the host range of ranaviruses could be determined by binding to other cellular proteins, binding to cell surface GAGs likely plays an important role in the initial interaction between virus and host cell.

It is worth noting that there are two types of virions for iridoviruses. One contains a central core surrounded by an internal membrane and a viral capsid. The other type has an outer viral envelope after the virions bud from the plasma membrane [9]. The two types of viral particles may have different cellular receptors when they infect cells. Thus, the existence of different virions that have an outer envelope or not could be another reason for the incomplete inhibition efficiency in the present study.

This is a report showing that iridoviruses bind cells of different species through the interaction between cell surface GAGs and envelope proteins. However, it remains to be determined whether binding to GAGs is both necessary and sufficient for subsequent virion entry, or whether virus—GAG interaction precedes binding to a second specific cellular receptor.
